# The Conflicting Role of Caffeine Supplementation on Hyperoxia-Induced Injury on the Cerebellar Granular Cell Neurogenesis of Newborn Rats

**DOI:** 10.1155/2022/5769784

**Published:** 2022-05-31

**Authors:** Vivien Giszas, Evelyn Strauß, Christoph Bührer, Stefanie Endesfelder

**Affiliations:** Department of Neonatology, Charité-Universitätsmedizin Berlin, Berlin, Germany

## Abstract

Preterm birth disrupts cerebellar development, which may be mediated by systemic oxidative stress that damages neuronal developmental stages. Impaired cerebellar neurogenesis affects several downstream targets important for cognition, emotion, and speech. In this study, we demonstrate that oxidative stress induced with high oxygen (80%) for three or five postnatal days (P3/P5) could significantly damage neurogenesis and proliferative capacity of granular cell precursor and Purkinje cells in rat pups. Reversal of cellular neuronal damage after recovery to room air (P15) was augmented by treatment with caffeine. However, downstream transcripts important for migration and differentiation of postmitotic granular cells were irreversibly reduced by hyperoxia, without rescue by caffeine. Protective effects of caffeine in the cerebellum were limited to neuronal survival but failed to restore important transcript signatures.

## 1. Introduction

Intensive care of extremely and very prematurely born preterm infants and consequently their survival have changed significantly over the past decades [1]. Preterm birth is one of the main risk factors for surviving with major and minor neurodevelopmental morbidities [2]. This incremental risk correlates with lower gestational age and birth weight [3]. In addition to the major neurodevelopmental morbidities, such as cerebral palsy and motor/cognitive delay, it is above all the supposedly minor neurodevelopmental abnormalities, including intellectual disability, emotional behavioral and neurobehavioral problems, and socioemotional difficulties like attention deficit hyperactivity disorder (ADHD) or autism spectrum disorder (ASD), that can be responsible for lifelong impairments [4–7].

Preterm birth *per se* as well as brain injuries such as fetal growth restriction [8], intraventricular hemorrhages [9], oxygen toxicity [10], neonatal stroke [11], or inflammatory events [12] may well interrupt brain development and maturation as independent risk factors or in combination and seem to be causative for adverse neuronal developmental disorders. There is a strong correlation between prematurity and a worse prognosis for the development of neurological impairments [13], but also for reduced regional brain volumes [14, 15]. Decreased cerebellar volumes and delayed growth are associated with noxious perinatal and postnatal factors [16, 17]. The cerebellar development of preterm infants and the crucial function of the cerebellum in terms of complex neurological abilities for cognitive and language development have gained more attention in recent years [18–21]. Impairments of complex functions have been associated with cerebellar injury, which then leads to deficits in language and cognitive skills, as well as altered social, emotional, and behavioral development of preterm infants [19, 22–24].

**Figure 1:**
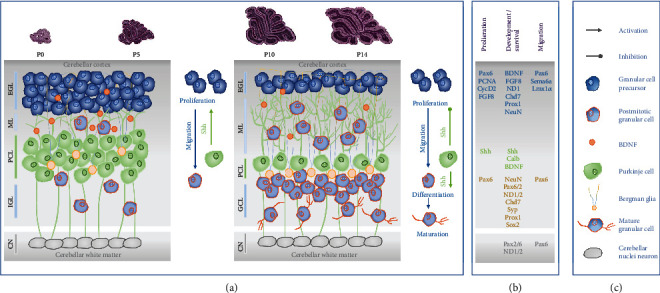
Schematic illustration of rodent postnatal granular cell neurogenesis. (a) The major cell type in the cerebellar cortex is the granule cell (GC). GC precursors (GCPs), originating from the rhombic lip, migrate to the mitotically active external granular layer (EGL). GCPs within the EGL are mitotically active from E15 until the third postnatal week. At the time of birth (P0), GCPs begin to differentiate into mature GCs (around P5) and reach their destination (at P20) by radial migration along the Bergmann glia of the GC into the internal granular layer (IGL) over the molecular layer (ML). In rats, the proliferation of cerebellar interneurons and granule cells that arise from the EGL lasts for over 3 weeks after birth. Proliferative activity of GCPs is regulated by extrinsic Purkinje cell- (PC-) derived factor sonic hedgehog (Shh). Brain-derived neurotrophic factor (BDNF), expressed in GCs, regulates survival and migration of GCPs and stimulates own migration of granule cells from EGL to IGL. (b) Cerebellar neurogenesis of granular cells is characterized by the expression of neuronal and proliferative markers and is orchestrated by neuronal transcription factors: brain-derived neurotrophic factor (BDNF), calbindin 1 (Calb1), chromodomain helicase DNA-binding protein 7 (Chd7), cyclin dependent kinase 2 (CycD2), fibroblast growth factor 8 (FGF8), hypoxanthine-guanine phosphoribosyl-transferase (HPRT), LIM homeobox transcription factor 1 alpha (Lmx1*α*), neuronal differentiation 1/2 (NeuroD 1/2), neuronal nuclei (NeuN), paired box 2/6 (Pax2/Pax6), proliferating cell nuclear antigen (PCNA), prospero homeobox 1 (Prox1), semaphoring 6a (Sema6a), sonic hedgehog signaling molecule (Shh), sex-determining region Y-box 2 (Sox2), and synaptophysin (Syp). (c) Cellular players in cerebellar granular cell neurogenesis.

The mature cerebellum consists anatomically of the vermis (median) and the two hemispheres (lateral). In the last trimester of pregnancy, the cerebellum undergoes fundamental changes in growth and volume increase during the brain growth spurt [25–27]. The most important first events in the development of the cerebellum in humans begin at about 24 weeks' gestation, with a growth peak at term birth and lasting until about the first year of life [26, 28]. In rodents, comparable cerebellar development occurs postnatally until the third week of life [29, 30]. Cerebellar development is characterized by formation of the cerebellar white matter, maturation of the Purkinje cell (PC) layer, maturation of mitotic granule cell (GC) precursors (GCPs) to postmitotic GCs, and their radial migration from external granular cell layer (EGL) along Bergman glial cell fibers to the formation of the internal granular cell layer (IGL) [31]. The cerebellar neurogenesis of granular cells is characterized by the expression of neuronal and proliferative markers and is orchestrated by neuronal transcription factors (see schematic illustration Figure 1).

Due to the long human cerebellar developmental period from the embryonic stage to the second year of life, but especially during the phase of rapid growth during the last third of pregnancy, children born too early may be exposed to a severe impairment of cerebellar development. Necessary intensive medical care and treatment of very preterm (28-32 weeks gestation) and extremely preterm (<28 weeks gestation) infants represent exogenous factors that can likewise sustainably interrupt neuronal cerebellar development [17, 20].

Preterm infants have frequently unstable respiration after birth. Arterial partial pressure of oxygen doubles during the intrauterine to extrauterine transition; additional oxygen supplementation enhanced arterial partial pressure of oxygen four- to fivefold, causing systemic oxidative stress [32]. Oxidative stress is a critical factor in proven injuries in the immature brain of the preterm infant, which correlates with neurodevelopmental abnormalities of prematurity [33, 34]. Normally, the antioxidant enzyme system can counteract oxidative stress well with a corresponding antioxidant response. However, the antioxidant defense system undergoes significant developmental changes during the neonatal period, resulting in significantly lower antioxidant cellular defense in preterm infants compared to term infants [35]. The therapeutic strategy of reducing the pathological effects of oxidative stress, such as oxidation of biomolecules, inflammation, or cell death [36], through antioxidative therapies, such as melatonin, surfactant, vitamin A, vitamin E, or caffeine, is not new [37].

One of the standard drugs most commonly used in neonatal care for the treatment of apnea in preterm infants is the methylxanthine caffeine. As a nonspecific inhibitor of adenosine receptor subtypes A1 and A2a, caffeine has a broad spectrum of pharmacokinetic activity [38, 39]. In addition to reducing the frequency of respiratory arrest, caffeine showed well-described short- and long-term effects [40]. Demonstrated neuroprotective effects in the developing brain reduced the rate of bronchopulmonary dysplasia (BPD) and death and likewise shortened the duration of mechanical ventilation, thus possibly reducing persistent oxidative stress [40–43]. When used within the first few days of life, the effects appear to be the most effective [44, 45]. As a potential free radical scavenger [46], as shown in experimental studies, caffeine could be an antioxidant *per se* [47, 48] and also has anti-inflammatory as well as antiapoptotic effects [49–51].

In the current study, we demonstrated that early exposure to high oxygen impairs cerebellar granular cell neurogenesis by analyzing newborn rats with cell type-specific markers. Based on previous experimental studies, we hypothesized that the antioxidant caffeine might minimize the effects of oxidative stress and found conflicting pleiotropy caffeine effects that require further discussion.

## 2. Materials and Methods

### 2.1. Animal Welfare

Time-pregnant *Wistar* rat dams were obtained from the Department of Experimental Medicine (FEM, Charité-Universitätsmedizin Berlin, Germany). The adult rats were housed in individual cages under environment-controlled conditions with a constant 12 h/12 h light/dark cycle, ambient temperature, and relative humidity of 60% with *ad libitum* access to the same food and water. After birth, the newborns were maintained with a mother for breast milk feeding. All animal experimental procedures were evaluated and approved by the local animal welfare authorities (LAGeSo, approval number G-0088/16) and followed institutional guidelines as well as ARRIVE guidelines.

### 2.2. Oxygen Exposure and Drug Administration

As previously described [47, 50], pups from different litters and both sexes were pooled and randomized within 12 h of birth and returned to the dams. Sample size calculation was performed in G∗Power V3.1.2 [52]. The newborn rats were randomly assigned to room air (normoxia, NO) or oxygen-enriched atmosphere (hyperoxia, HY) treatment. The pups in hyperoxia subgroups were reared with the dams in an atmosphere containing 80% oxygen (OxyCycler BioSpherix, Lacona, NY) from postnatal day (P)0 to P3 (*n* = 6-8) or P0 to P5 (*n* = 6*-*8); in parallel, the pups in normoxia groups were reared with the dams under room air conditions. To avoid oxygen toxicity in the nursing mothers, they were rotated between the hyperoxic and normoxic litters every 24 h. The rats were divided into four groups, each for same exposure times to (i) normoxia (NO, control group): 21% oxygen application of vehicle (phosphate-buffered saline, PBS), (ii) normoxia with caffeine (NOC): 21% oxygen with caffeine (10 mg/kg, Sigma, Steinheim, Germany), (iii) hyperoxia (HY): 80% oxygen with vehicle (PBS), and (iv) hyperoxia with caffeine (HYC): 80% oxygen with caffeine (10 mg/kg). 10 mg/kg of pure caffeine is equivalent to 20 mg/kg caffeine citrate, which is used clinically [53]. Rat pups received either caffeine or vehicle injection intraperitoneally (i.p.) as a fixed proportion of their body weight (100 *μ*l/10 g) every 48 h beginning on the day of birth (P0). Caffeine or vehicle was administered to the pups with a total of three postnatal days of oxygen exposure (P0-P3) on the day of birth (P0) and on P2; and for the rat pups with a total of five days of postnatal oxygen exposure (P0-P5) on the day of birth (P0) and on P2 and P4. The rat pups were examined after the oxygen exposure (P3, P5) either directly or after recovery in room air at P15 (P3_P15, P5_P15). No pups died during hyperoxia. Caffeine plasma concentrations and weight profiles were determined and presented in previous work [47].

### 2.3. Tissue Preparation

At the experimental endpoints (P3; P3_P15; and P5; P5_P15), rat pups were anaesthetized with an i.p. injection of ketamine (100 mg/kg), xylazine (20 mg/kg), and acepromazine (3 mg/kg) and then transcardially perfused, as previously described [47, 50]. In deep sedation and after perfusion, the rats were decapitated in the cervical region. The whole brain tissues were excised and processed for molecular analysis or histology. For the gene expression studies, cerebellar tissues were snap-frozen in liquid nitrogen and stored at -80°C. The perfusion was carried out with PBS (pH 7.4). For immunohistochemical analysis, the PBS perfusion was followed by perfusion with 4% paraformaldehyde (pH 7.4); the cerebellums were postfixed at 4°C for 1 day, embedded in paraffin, and processed for histological staining.

### 2.4. RNA Extraction and Quantitative Real-Time PCR

Tissue procurement has already been described [50]. Briefly, total RNA was isolated from one snap-frozen cerebellar hemisphere per animal by acidic phenol/chloroform extraction (peqGOLD RNAPure™; PEQLAB Biotechnologie, Erlangen, Germany) and 2 *μ*g of RNA was DNase treated and reverse-transcribed. The PCR products of *brain-derived neurotrophic factor* (*BDNF*), *Calbindin 1* (*Calb1*), *chromodomain helicase DNA-binding protein 7* (*Chd7*), *cyclin dependent kinase 2* (*CycD2*), *fibroblast growth factor 8* (*FGF8*), *hypoxanthine-guanine phosphoribosyl-transferase* (*HPRT*), *LIM homeobox transcription factor 1 alpha* (*Lmx1α*), *neuronal differentiation 1/2* (*NeuroD 1/2*), *neuronal nuclei* (*NeuN*), *paired box 2/6* (*Pax2/Pax6*), *prospero homeobox 1* (*Prox1*), *semaphoring 6a* (*Sema6a*), *sonic hedgehog signaling molecule* (*Shh*), *sex-determining region Y-box 2* (*Sox2*), and *synaptophysin* (*Syp*) were quantified in real time with the sequences summarized in Table 1. PCR and detection were performed with qPCRBIO Mix Hi-ROX (NIPPON Genetics Europe, Düren, Germany) with HPRT used as an internal reference. The detection of PCR products was performed in triplicate in 11 *μ*l reaction mix, each containing 5 *μ*l of qPCR mastermix, 2.5 *μ*l of 1.25 *μ*M of each oligonucleotide primer, 0.5 *μ*l of 5 *μ*M of probe, and 3 *μ*l of cDNA template (17 ng). PCR amplification was performed in 96-well reaction plates for 40 cycles, each cycle at 94°C for 5 s and 62°C for 25 s. The expression of target genes was analyzed with the StepOnePlus real-time PCR system (Applied Biosystems, Carlsbad, CA, USA) according to the 2^-*ΔΔ*CT^ method [54].

### 2.5. Immunohistochemistry

Paraffin-embedded cerebellar sections were serially cut into 6 *μ*m sections and mounted onto Superfrost Plus-coated slides (Menzel, Braunschweig, Germany). The sections, as previously described [55], were deparaffinized in Roti-Histol (Carl Roth, Karlsruhe, Germany) twice for 10 min each. The PFA-fixed tissues were dehydrated through incubation in aqueous solutions of decreasing ethanol concentration. The slices were subsequently hydrated in ethanol (100%, 100%, 90%, 80%, and 70%) for 3 min each. To demask intracellular epitopes, sections were fixed in citrate buffer (pH 6.0) in a microwave oven for 10 min at 600 W. All slides were then cooled at room temperature for half an hour before being washed three times in PBS. For calbindin/DAPI staining blocking solution, 3% bovine serum albumin (BSA), 0.05% TW-20, and 0.1% Triton X-100 in PBS were applied to each section for 60 min at room temperature. For Pax6/PCNA/DAPI staining, blocking solution 3% BSA and 0.2% Triton X-100 in PBS were used instead. Primary antibodies were applied after washing once with PBS; slides were incubated overnight at 4°C with either monoclonal mouse anti-calbindin (1 : 500, Abcam, ab75524) or polyclonal rabbit anti-Pax6 (1 : 200, LifeSpan Bioscience, LS-C179903) with monoclonal mouse anti-PCNA (1 : 500, Abcam, ab29) diluted in antibody diluent (Zymed Laboratories, San Francisco, CA). Slices were washed in PBS three times, and secondary Alexa Fluor 488-conjugated goat anti-mouse IgG (Thermo Fisher Scientific, Dreieich, Germany) or Alexa Fluor 594-conjugated goat-anti-rabbit IgG (Thermo Fisher Scientific) was applied, with consistent 1 : 200 dilution in antibody diluent (Zymed Laboratories). Sections were incubated for 1 h at room temperature, were consecutively washed once in PBS, and were incubated for 10 min at room temperature after applying 4′,6-diamidino-2-phenylindole (DAPI, Sigma) diluted 1 : 2000 in PBS for counterstaining. We mounted all sections after three final washes with PBS (Shandon Immu-Mount, Thermo Fisher Scientific).

Midsagittal cerebellar sections were analyzed blind using a Keyence compact fluorescent microscope BZ 9000 with BZ-II Viewer software and BZ-II Analyzer software (Keyence, Osaka, Japan) using 10x objective lenses and individual files stitched automatically for each RGB color. Pictures were taken with the same exposure time and contrast/brightness parameters. For analysis, four nonoverlapping separate images of posterior lobules IV/V, VI, and/or VII per animal were obtained, including at least two external cortices and two inner loops of the cerebellar cortex. For quantification of Purkinje cells (calbindin+), granule neuron (Pax6), and proliferation marker (PCNA), four 100 *μ*m (P3 and P5) or 200 *μ*m (P3_P15 and P5_P15) regions of lobules were quantified for each section and were counted manually using Adobe Photoshop software 22.0.0 (Adobe Systems Software Ireland Limited, Dublin, Republic of Ireland) with minimal previous manipulation of contrast. The molecular layer thickness was determined on identical digital sections of calbindin count images, and the dendrite length of Purkinje cells was used to evaluate the molecular layer thickness by measuring the primary dendrite from the soma up to the surface of the molecular layer. The values determined in this way of cell counts and molecular layer thickness ensured a more representative result across the whole lobule. DAPI was used to visualize the cell nucleus and to mark the granular layer. Mean values per sample were calculated by averaging the values of all sections of the same animal and were used to compare the cell counts and layer thickness of treated animals versus control animals.

### 2.6. Statistical Analyses

Box and whisker plots represent the interquartile range (box) with the line representing the median, while whiskers show the data variability outside the upper and lower quartiles. As previously described [50], groups were compared using one-way analysis of variance (ANOVA), based on a partially non-Gaussian distribution with the Kruskal-Wallis test or based on the assumption that groups do not have equal variances with the Brown-Forsythe test. Depending on which ANOVA test was used, multiple comparisons of means were carried out using Bonferroni's, Dunn's, or Dunnett's T3 *post hoc* test. A *p* value of <0.05 was considered significant. All graphics and statistical analyses were performed using the GraphPad Prism 8.0 software (GraphPad Software, La Jolla, CA, USA).

## 3. Results

### 3.1. Caffeine Reverses Hyperoxia-Induced Impairment of Purkinje Cells

**Figure 2:**
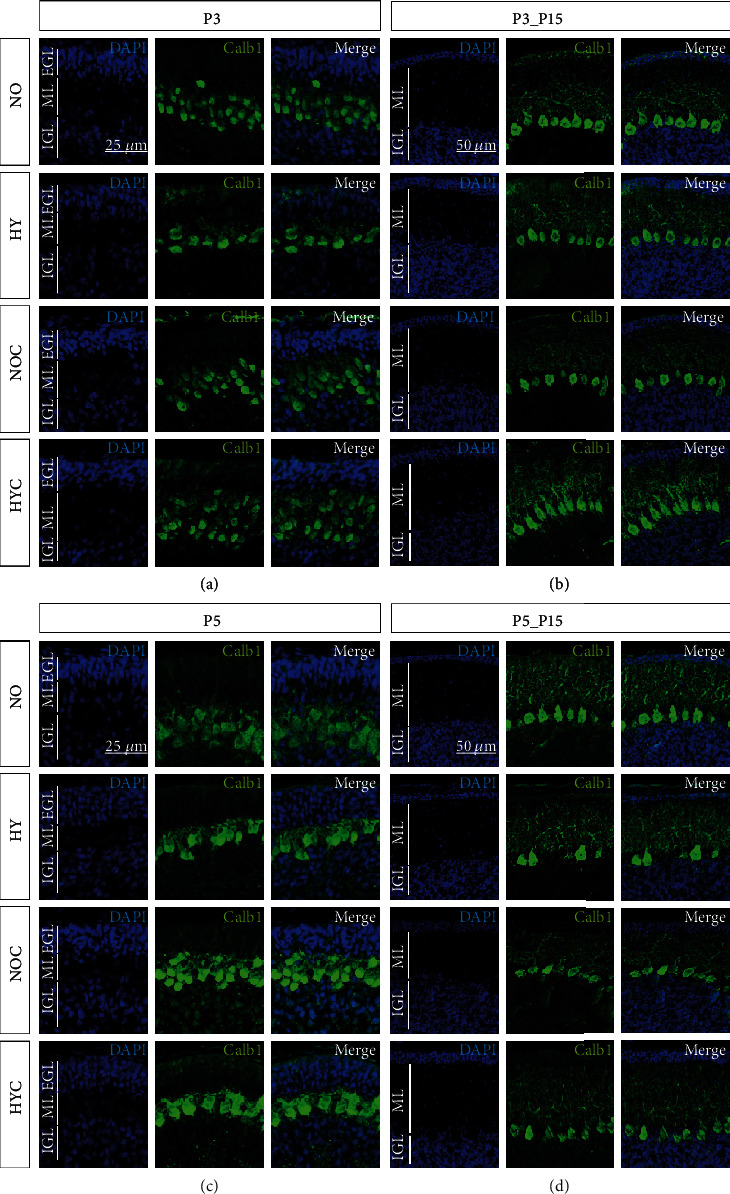
Representative cerebellar paraffin sections colabeled with calbindin and DAPI of rat pups exposed to normoxia (NO) or hyperoxia (HY) compared to rat pups treated with caffeine (NOC, HYC). Examinations were performed at postnatal day 3 (P3 (a)) and P5 (c) or after recovery after 3-day exposure at P15 (b) or after 5-day exposure at P15 (d). Immunofluorescent images indicated calbindin (green) and nuclei (blue, DAPI). Three- (P3) and five-day (P5) lasting hyperoxia affects the density and length of dendrites of Purkinje cells in the newborn rat cerebellum and persists mainly after 5 day hyperoxia exposure until P15 (P5_P15). Caffeine was found to partly counteract these changes. EGL: external granular layer; ML: molecular layer; IGL: internal granular layer. Scale bar P3/P5 with 25 *μ*m and P3_P15/P5_P15 with 50 *μ*m.

**Figure 3:**
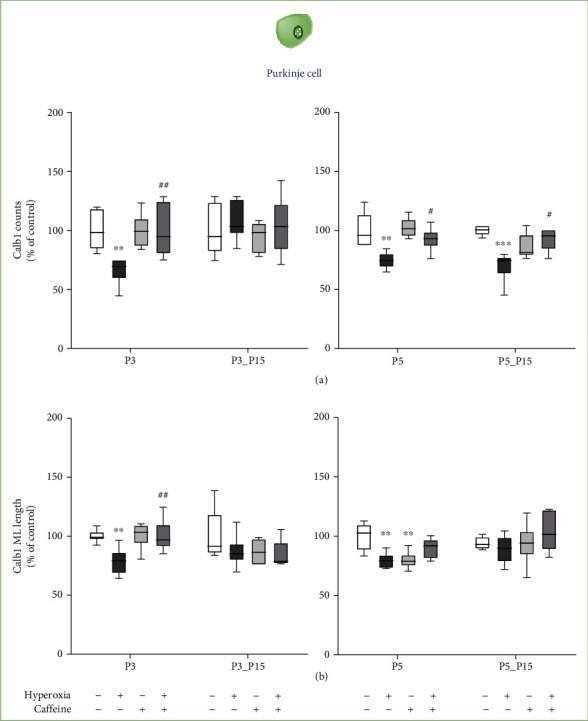
Quantification of (a) counts of colabeled calbindin and DAPI-positive Purkinje cells (PC) of the cerebellar molecular layer and (b) molecular layer (ML) length was performed for 3 days' postnatal oxygen exposure (P3) and recovery (P3_P15) and 5 days' postnatal oxygen exposure (P5) and recovery (P5_P15), respectively. Acute hyperoxia exposure (deep dark gray bars) decreased the number of Purkinje cells and depressed the dendrite length. Caffeine administration (gray bars) was a protective effect for calbindin-positive cells, but not for dendrite growth. Caffeine with normoxia (light gray bars) did not influence density and dendrite length of Purkinje cells, except at P5. Data are normalized to the level of rat pups exposed to normoxia at each time point (control 100%, white bars), and the 100% values are 1.4 (P3), 2.8 (P3_P15), 1.4 (P5), and 2.5 (P5_P15) length of the molecular layer or 14 (P3), 7.4 (P3_P15), 8.9 (P5), and 7.2 (P5_P15) cells per regions of lobules, respectively. *n* = 6-8/group. ^∗^*p* < 0.05, ^∗∗^*p* < 0.01, ^∗∗∗^*p* < 0.001, and ^∗∗∗∗^*p* < 0.0001 vs. control; ^#^*p* < 0.05, ^##^*p* < 0.01, and ^###^*p* < 0.001 vs. hyperoxia (ANOVA, Bonferroni's *post hoc* test; Kruskal-Wallis, Dunn's *post hoc* test).

In this experiment, we investigated the changes in the amount of calbindin-positive Purkinje cells and the length of their dendrites in the molecular layer (ML) after exposure to hyperoxia and application of caffeine (Figures 2 and 3). Additionally, cerebellar expression of calbindin (*Calb1*) and *NeuN* was quantified using qRT-PCR (Figure 3). Hyperoxia significantly reduced counts of calbindin-positive Purkinje cells and dendrite length at P3 and P5 compared to normoxic control litters (Figures 2(a), 2(c), 3(a) and 3(b)). After the hyperoxic insult, cell counts and dendrite length decreased at P3 and/or P5 (Figures 3(a) and 3(b)). At P3, caffeine was able to attenuate this effect and maintain cell counts and dendrite growth (Figures 3(a) and 3(b)). At P15 after recovery in normoxic environment, no significant disparities between groups were measurable, besides the calbindin cell counts after five-day hyperoxia at P15 (P5_15 group), which did not recover from the hyperoxic insult. The complete data are shown in full in a supplementary table (see Table S-1).

Caffeine administered during early high oxygen exposure counteracted the reduced number of Purkinje cells and, to a lesser extent during three-day exposure, their shortened dendrites.

### 3.2. Caffeine Rescues Pax6-Positive Granule Cells and Proliferation Capacity Damaged by Short-Term Hyperoxia

**Figure 4:**
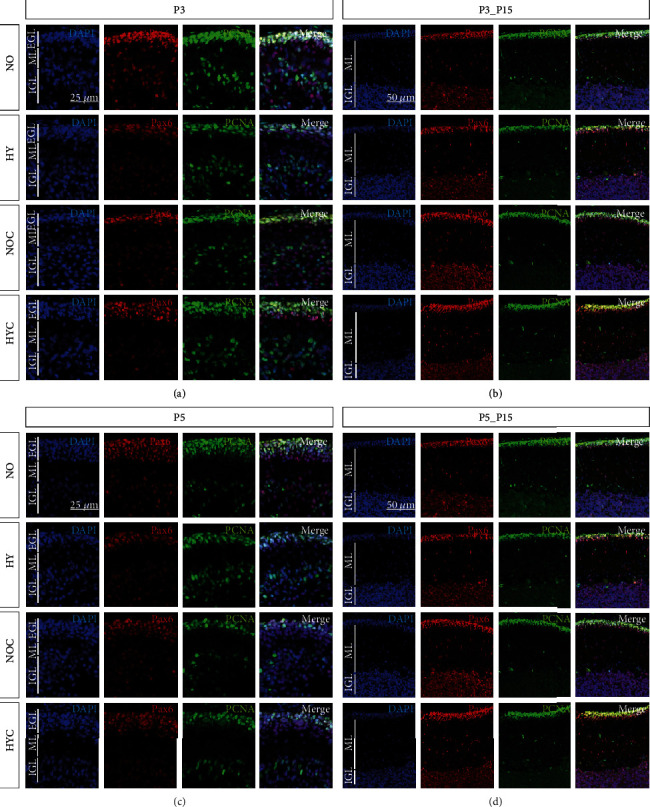
Representative cerebellar paraffin sections colabeled with Pax6, PCNA, and DAPI of rat pups exposed to normoxia (NO) or hyperoxia (HY) compared to rat pups treated with caffeine (NOC, HYC). Examinations were performed at postnatal day 3 (P3 (a)) and P5 (c) or after recovery after 3-day exposure at P15 (b) or after 5-day exposure at P15 (d). Immunofluorescent images indicated Pax6 (red), PCNA (green), and nuclei (blue, DAPI). Three -(P3) and five-day (P5) lasting hyperoxia affected the density of granule cells (Pax6), proliferation capacity (PCNA), and thickness of EGL in the newborn rat cerebellum after acute hyperoxia exposure (P3, P5). Caffeine at P3 was partly effective to inhibit the effects of hyperoxia. EGL: external granular layer; ML: molecular layer; IGL: internal granular layer. Scale bar P3/P5 25 *μ*m and P3_P15/P5_P15 50 *μ*m.

**Figure 5:**
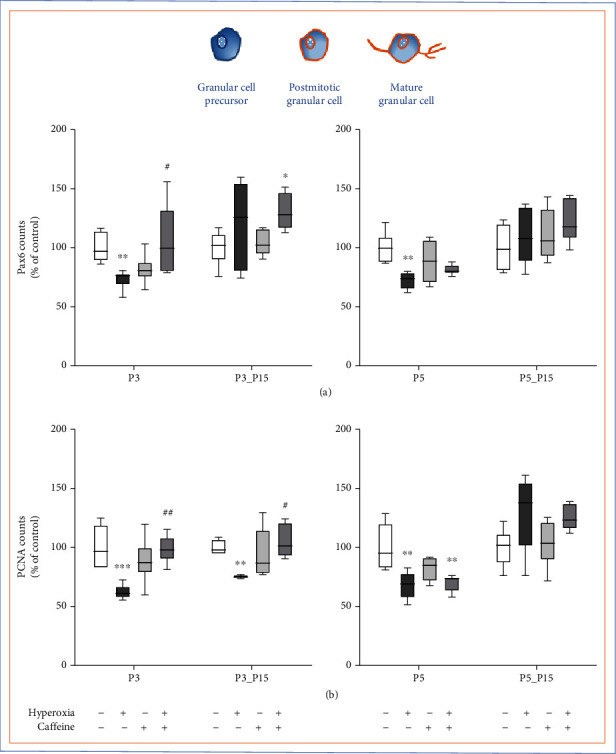
Quantification of (a) Pax6 and (b) PCNA counts of colabelled positive cells of the cerebellar molecular layer was performed for 3 days' postnatal oxygen exposure (P3) and recovery (P3_P15) and 5 days' postnatal oxygen exposure (P5) and recovery (P5_P15), respectively. Acute hyperoxia exposure (P3 and P5; deep dark gray bars) decreased the number of granular cells (Pax6) and proliferating cells (PCNA). Caffeine (gray bars) was protective for Pax6-positive cells at P3 and P5 and for proliferating capacity at P3 with recovery until P15 (P3_P15). Caffeine with normoxia (light gray bars) did not influence density of granular cells and proliferation. Data are normalized to the level of rat pups exposed to normoxia at each time point (control 100%, white bars), and the 100% values are 38 (P3), 54.4 (P3_P15), 46.6 (P5), and 41.3 (P5_P15) Pax6+ cells per regions of lobules or 33 (P3), 37.1 (P3_P15), 65.7 (P5), and 22.3 (P5_P15) PCNA+ cells per region of lobules, respectively. *n* = 6-8/group. ^∗^*p* < 0.05, ^∗∗^*p* < 0.01, and ^∗∗∗^*p* < 0.001 vs. control; ^#^*p* < 0.05 and ^##^*p* < 0.01 vs. hyperoxia (ANOVA, Bonferroni's *post hoc* test; Kruskal-Wallis, Dunn's *post hoc* test; Brown-Forsythe, Dunnett's *post hoc* test).

**Figure 6:**
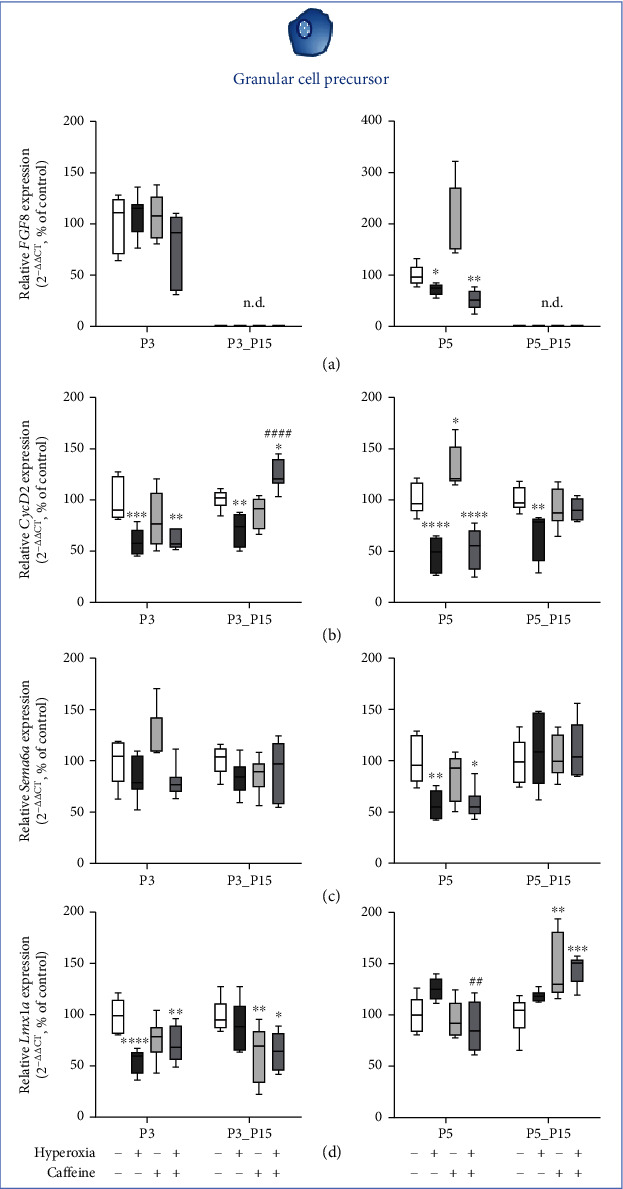
Quantification of cerebellar homogenates for granule cell precursor-associated mediators of (a) *FGF8*, (b) *CycD2*, (c) *Sema6a*, and (d) *Lmx1α* was performed with qPCR for 3 days' postnatal oxygen exposure (P3) and recovery (P3_P15) and 5 days' postnatal oxygen exposure (P5) and recovery (P5_P15), respectively. Acute hyperoxia exposure (deep dark gray bars) decreased the mRNA expression at P3 and/or P5. At P15, only the reduction of the proliferation marker *CycD2* persisted. Caffeine failed to reverse the hyperoxia-induced effects (gray bars), except for *CycD2* at P3_P15. Caffeine with normoxia (light gray bars) modulated the expression levels in a more differential manner. Data are normalized to the level of rat pups exposed to normoxia at each time point (control 100%, white bars). *n* = 6-8/group. ^∗^*p* < 0.05, ^∗∗^*p* < 0.01, ^∗∗∗^*p* < 0.001, and ^∗∗∗∗^*p* < 0.0001 vs. control; ^##^*p* < 0.01 and ^####^*p* < 0.0001 vs. hyperoxia (ANOVA, Bonferroni's *post hoc* test; Kruskal-Wallis, Dunn's *post hoc* test; Brown-Forsythe, Dunnett's *post hoc* test).

We investigated the changes of Pax6-positive GCP counts migrating as postmitotic GCs in the ML and proliferation capacity after exposure to hyperoxia with or without application of caffeine (Figures 4–6(a) and 6(b)). As can be clearly seen in optical density (Figure 4) and after quantification (Figure 5), at P3 and P5, Pax6- as well as PCNA-positive cells presented a significant quantitative decrease after exposure to 80% hyperoxia. Pax6 cells as well as PCNA-positive cells were reduced at P3 and P5 (Figures 5(a) and 5(b)) compared to the control group in room air. At P3, caffeine significantly attenuated the negative effect of oxidative stress and maintained cell counts similar to those of control animals for PCNA. Caffeine had no protective effect on Pax6-expressing cells or the number of proliferating cells after 5 days of oxygen exposure (Figures 5(a) and 5(b)). For those groups at P15 following recovery at atmospheric air, at P5_P15, no significant variations between groups were measurable (Figure 5), whereas at P3_P15, Pax6-positive cell counts were significantly increased (Figure 5(a)) compared to control groups. PCNA-positive cell counts were also significantly elevated (Figure 5(b)) compared to the hyperoxic group without caffeine application. The complete data are shown in full in a supplementary table (see Table S-2).

The three-day hyperoxia-induced decreased proliferative capacity of mainly progenitors, which persisted after recovery from oxygen toxicity, was reversed by caffeine at early exposure.

### 3.3. Caffeine Administration Fails to Rescue Granular Cell Progenitor Impairment Mediated by Acute Hyperoxia

Gene expression of *FGF8* and *CyclinD2* was significantly decreased after acute exposure to hyperoxia at P3 and/or P5. Caffeine had no protective effect to mitigate hyperoxia-mediated injury (Figures 6(a) and 6(b)). A reduction of transcription under hyperoxic damage occurred for *FGF8* (Figure 6(a)) at P5 and *CyclinD2* (Figure 6(b)) at P3 and P5, respectively. Caffeine under hyperoxic exposure decreased expression of *CyclinD2* (Figure 6(b)) at P3 and for *FGF8* (Figure 6(a)) and *CyclinD2* (Figure 6(b)) at P5. Decreased expression after hyperoxic injury persisted after recovery until postnatal day 15 (P15) only for *Cyclin D2* at P3_P15 and at P5_P15 (Figure 6(b)), respectively. Caffeine was not able to immediately attenuate the oxidative stress, except for *CyclinD2*. Animals that experienced hyperoxia after application of caffeine even showed higher expression levels (Figure 6(b)). FGF8 plays an important role in early developmental stages and structural polarity prior to cerebellar specification [28], and in conclusion, no expression was detected at P15 time points. The complete data are shown in full in a supplementary table (see Table S-3).

GCP-associated cerebral developmental factors for proliferation and migration were strongly affected by acute hyperoxia, whereas caffeine only restored the proliferation marker after recovery to room air.

### 3.4. Caffeine Does Not Alter Morphogen and Neurotrophin Granular Cell Progenitor-Associated Downregulation Caused by Acute Hyperoxia

**Figure 7:**
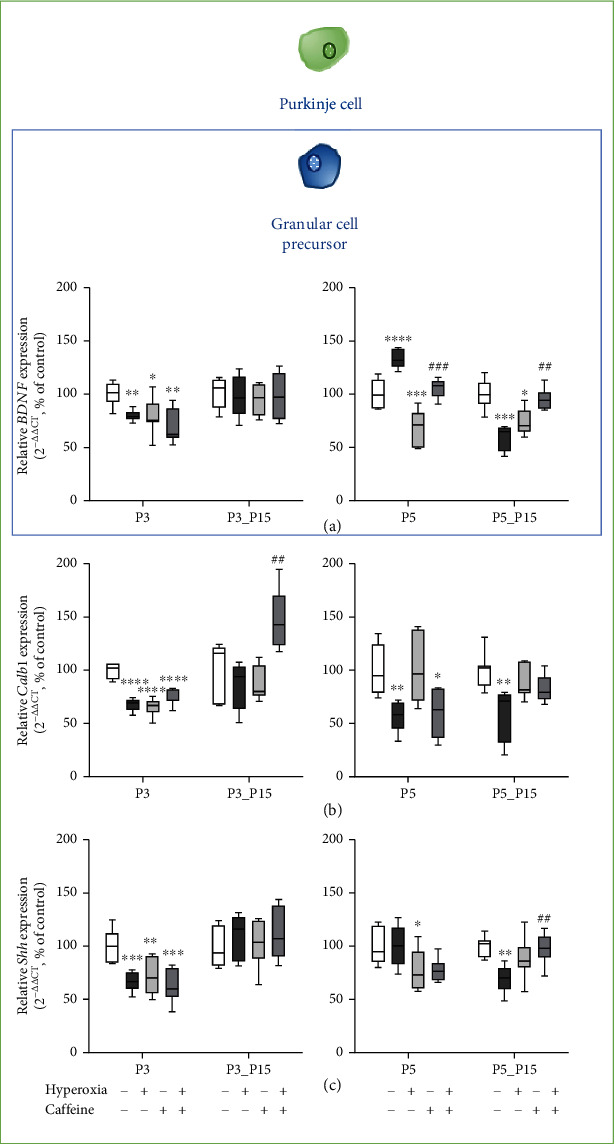
Quantification of cerebellar homogenates for granule cell precursor- and/or Purkinje cell-associated mediators of (a) *BDNF*, (b) *Calb1*, and (c) *Shh* was performed with qPCR for 3 days' postnatal oxygen exposure (P3) and recovery (P3_P15) and 5 days' postnatal oxygen exposure (P5) and recovery (P5_P15), respectively. Acute hyperoxia exposure (deep dark gray bars) decreased the mRNA expression at P3 and/or P5, except for *BDNF* with an increase at P5. At P15, the reduction of *Calb1* persisted after three-day oxygen exposure (P3_P15), or the reduction of *BDNF*, *Calb1*, and *Shh* evolved after five-day oxygen exposure (P5_P15). Caffeine most antagonized hyperoxia-induced effects (gray bars) after recovery for *BDNF* and *Shh* at P5_P15 but reduced the overexpressed levels of *BDNF* at P5. Caffeine with normoxia (light gray bars) modulated the expression levels in a more differential manner. Data are normalized to the level of rat pups exposed to normoxia at each time point (control 100%, white bars). *n* = 6-8/group. ^∗^*p* < 0.05, ^∗∗^*p* < 0.01, ∗∗∗*p* < 0.001, and ^∗∗∗∗^*p* < 0.0001 vs. control;^##^*p* < 0.01 and ^###^*p* < 0.001 vs. hyperoxia (ANOVA, Bonferroni's *post hoc* test; Kruskal-Wallis, Dunn's *post hoc* test; Brown-Forsythe, Dunnett's *post hoc* test).

Gene expression of *BDNF* (Figure 7(a)) and *Shh* (Figure 7(c)) was significantly decreased after acute exposure to hyperoxia at P3, as well as gene expression of *Calb1* at P3 and P5 (Figure 7(b)). Three-day hyperoxia did not persist after recovery to room air, whereas five-day exposure to oxygen (P5_P15) significantly decreased *BDNF* (Figure 7(a)) and *Shh* (Figure 7(c)) expression after 10 days of recovery and continued to suppress *Calb1* expression (Figure 7(b)).

No protective caffeine effect to mitigate hyperoxia injury was detected at P3 or P5, but after recovery at P15 and high oxygen exposure for five postnatal days (P5_P15), increased caffeine gene expression of *BDNF* and *Shh* (Figures 7(a) and 7(c)) was found. Caffeine under normoxic exposure decreased significant expression of *BDNF* (Figure 7(a)), *Calb1* (Figure 7(b)), and *Shh* (Figure 7(c)) at P3 and for *BDNF* (Figure 7(a)) and *Shh* (Figure 7(c)) at P5, respectively. The complete data are shown in full in a supplementary table (see Table S-4).

Hyperoxic insult significantly reduced GPC-relevant transcripts of the neurotrophin BDNF and the mitogen Shh and could be protected by caffeine exclusively after recovery in room air after five days of hyperoxia.

### 3.5. Caffeine Has Only Minimal Effects on Hyperoxia-Injured Mediators of Cerebral Development of Mitotic and Postmitotic Granular Cells

**Figure 8:**
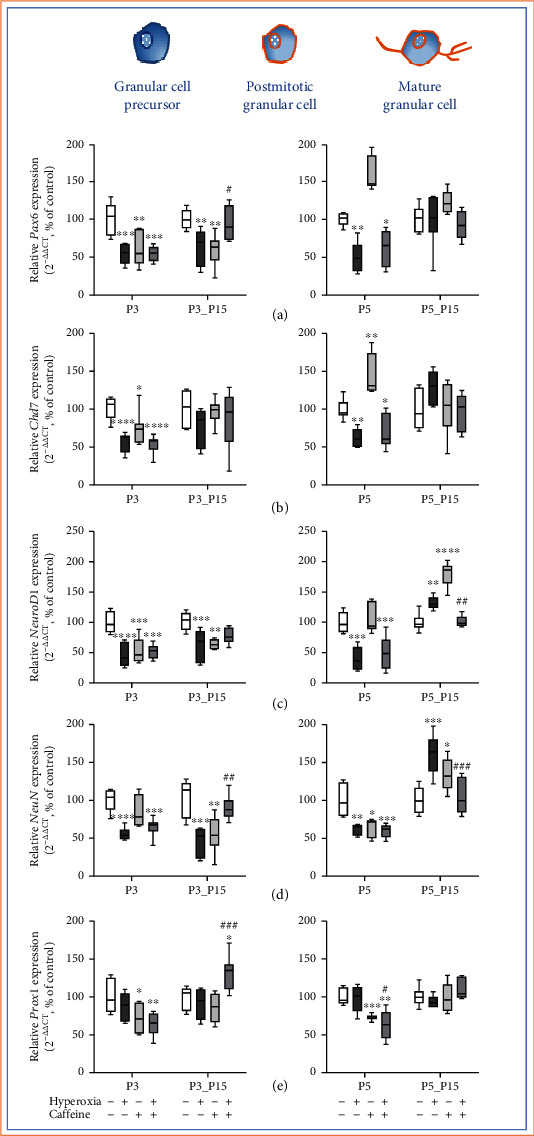
Quantification of cerebellar homogenates for granule cell precursor- and granule cell-associated mediators of (a) *Pax6*, (b) *Chd7*, (c) *NeuroD1*, (d) *NeuN*, and (e) *Prox1* was performed with qPCR for 3 days' postnatal oxygen exposure (P3) and recovery (P3_P15) and 5 days' postnatal oxygen exposure (P5) and recovery (P5_P15), respectively. Acute hyperoxia exposure (deep dark gray bars) decreased the mRNA expression at P3 and P5, except for *Prox1*. At P15, the reduction of *Pax6*, *NeuroD1*, and *NeuN* persisted after three-day oxygen exposure (P3_P15), as did the increased expression *NeuroD1* and *NeuN* after five-day oxygen exposure (P5_P15). Caffeine rescued the transcription in comparison to hyperoxia-exposed group (gray bars) for *Pax6*, *NeuN*, and *Prox1* at P3_15. Caffeine with normoxia (light gray bars) modulated the expression levels in a more differential manner. Data are normalized to the level of rat pups exposed to normoxia at each time point (control 100%, white bars). *n* = 6-8/group. ^∗^*p* < 0.05, ^∗∗^*p* < 0.01, ^∗∗∗^*p* < 0.001, and ^∗∗∗∗^*p* < 0.0001 vs. control; ^#^*p* < 0.05, ^##^*p* < 0.01, and ^###^*p* < 0.001 vs. hyperoxia (ANOVA, Bonferroni's *post hoc* test; Kruskal-Wallis, Dunn's *post hoc* test; Brown-Forsythe, Dunnett's *post hoc* test).

High oxygen exposures for the first three or five days of life significantly reduced mRNA transcription of *Pax6* (Figure 8(a)), of *Chd7* (Figure 8(b)), and of *NeuroD1* (Figure 8(c)), as well as *NeuN* (Figure 8(d)) at P3 and/or P5. This decreased gene expression persisted after recovery to room air for the three-day exposure period for *Pax6* (Figure 8(a)), for *NeuroD1* (Figure 8(c)), and for *NeuN* (Figure 8(d)) at P3_P15. After five days of oxygen exposure at P5_P15, expression increased significantly for *NeuroD1* (Figure 8(c)) and for *NeuN* (Figure 8(d)). Caffeine did change the effects after acute exposures at P3 and P5 but induced higher expression of *Pax6* (Figure 8(a)), of *NeuN* (Figure 8(d)), and of *Prox1* (Figure 8(e)) at P3_P15 and counteracted with lower transcription levels at P5_P15 (Figures 8(c) and 8(d)). Multiple administration of caffeine under control conditions also decreased expression at P3 for *Pax6* (Figure 8(a)), for *Chd7* (Figure 8(b)), for *NeuroD1* (Figure 8(c)), and for *Prox1* (Figure 8(e)) at P3, as well as for *NeuN* (Figure 8(d)) and *Prox1* (Figure 8(e)) at P5. This inhibition of transcription with caffeine persisted for *Pax6* (Figure 8(a)), for *NeuroD1* (Figure 8(c)), and for *NeuN* (Figure 8(d)) at P3_P15. Interestingly, under normoxia, caffeine induced the expression of *Pax6* (Figure 8(a)) and *Chd7* (Figure 8(b)) at P5, as well as *NeuroD1* (Figure 8(c)) and *NeuN* (Figure 8(d)) at P5_P15. The complete data are shown in full in a supplementary table (see Table S-5).

Neuronal factors for granular cell development and survival were markedly downregulated by oxygen toxicity, with protective caffeine effects detectable, if at all, after recovery to room air.

### 3.6. Caffeine Does Not Affect the Hyperoxia-Impaired Expression of Developmental Mediators of Migrating and Mature Granule Cells

**Figure 9:**
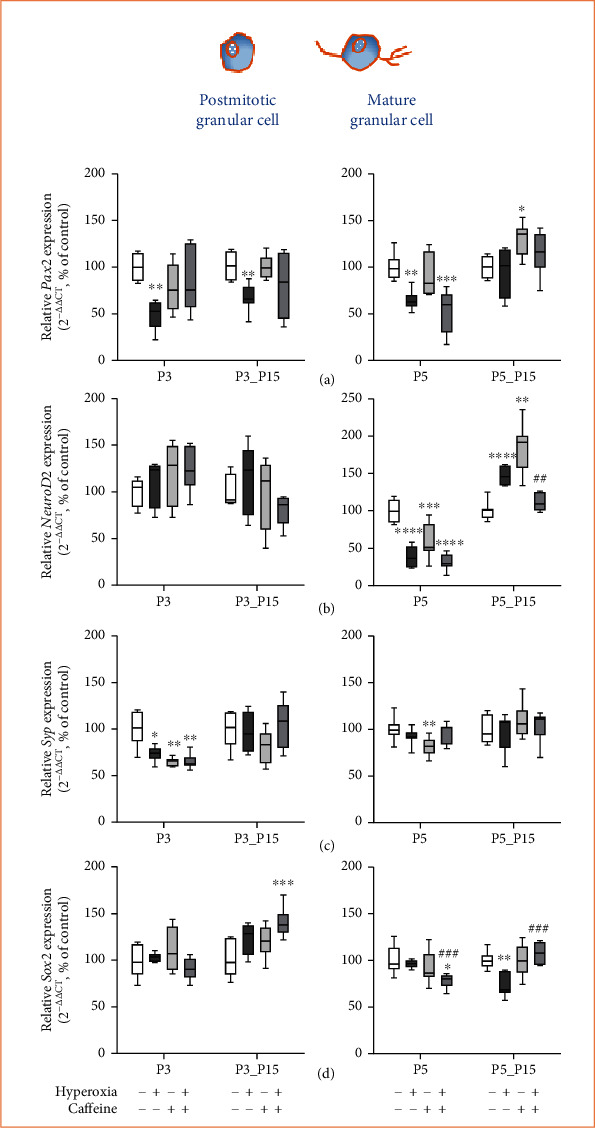
Quantification of cerebellar homogenates for postmitotic and mature granule cell-associated mediators of (a) *Pax2*, (b) *NeuroD2*, (c) *Syp*, and (d) *Sox2* was performed with qPCR for 3 days' postnatal oxygen exposure (P3) and recovery (P3_P15) and 5 days' postnatal oxygen exposure (P5) and recovery (P5_P15), respectively. Acute hyperoxia exposure (deep dark gray bars) decreased the mRNA expression for Pax2 and Syp at P3 and for Pax2 and NeuroD2 at P5. At P15, the reduction of *Pax2* persisted after three-day oxygen exposure (P3_P15), evolved after five-day oxygen exposure for Sox2 (P5_P15) or the expression increased of *NeuroD2* (P5_P15). Caffeine reverted the altered transcription in comparison to hyperoxia-exposed group (gray bars) for *NeuroD2* and *Sox2* at P5_15. Caffeine with normoxia (light gray bars) modulated the expression levels in a more differential manner. Data are normalized to the level of rat pups exposed to normoxia at each time point (control 100%, white bars). n =6-8/group. ^∗^*p* < 0.05, ^∗∗^*p* < 0.01, ^∗∗∗^*p* < 0.001, and ^∗∗∗∗^*p* < 0.0001 vs. control; ^##^*p* < 0.01 and ^###^*p* < 0.001 vs. hyperoxia (ANOVA, Bonferroni's *post hoc* test; Kruskal-Wallis, Dunn's *post hoc* test; Brown-Forsythe, Dunnett's *post hoc* test).

*Pax2* mRNA transcription is reduced under hyperoxic conditions after acute hyperoxia at P3 and P5 and persisted until P3_P15 (Figure 9(a)). *NeuroD2* was unaffected by three-day postnatal exposure, but five-day oxygen treatment reduced *NeuroD2* and resulted in aberrant expression (Figure 9(b)) after recovery in room air (P5_P15). High oxygen reduced *Syp* transcription only at P3 (Figure 9(c)), whereas *Sox2* showed downregulation only at P5_P15 (Figure 9(d)). Caffeine under hyperoxic impact did not show counteracting effects but also showed inhibitory effects similar to oxygen alone (*Syp* at P3 (Figure 9(c)), *Pax2* (Figure 9(a)), and *NeuroD2* (Figure 9(b)) at P5) or increased gene expression for *Sox2* at P3_P15 (Figure 9(d)) and P5_P15 (Figure 9(d)) or decreased for *NeuroD2* (Figure 9(b)) at P5_P15. Immediately after multiple applications of caffeine under normoxic conditions, transcription of *Syp* reduced at P3 (Figure 9(c)) and of *NeuroD2* (Figure 9(b)) and *Syp* (Figure 9(c)) at P5, while enhanced *Pax2* (Figure 9(a)) and *NeuroD2* was found at P5_P15 (Figure 9(b)). The complete data are shown in full in a supplementary table (see Table S-6).

Hyperoxia, acute as well as after recovery under normoxia, reduced neuronal factors for further differentiation and survival of mature granule cells, whereas no protective effect of caffeine was detectable.

## 4. Discussion

**Figure 10:**
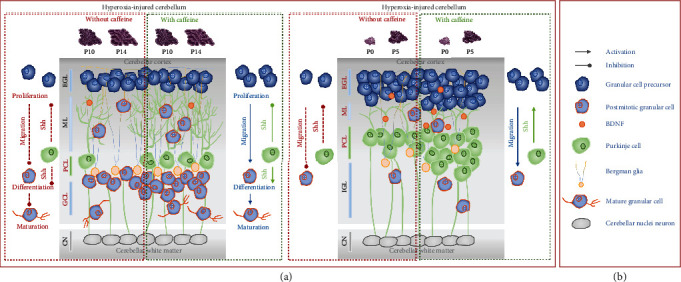
Schematic illustration of hyperoxia-injured rodent postnatal granular cell neurogenesis with and without caffeine treatment. (a) Hyperoxia reduced granule cell precursors (GPC) and Purkinje cells (PC) in the external granular layer (EGL) and Purkinje cell layer (PCL), respectively (up to P5, dashed red left box). Due to the influence of the high oxygen concentration, the dendrites of the PC were reduced and this resulted in a thinner molecular layer (ML), also after acute hyperoxia. The morphological changes persisted even after recovery in room air until the end of the second week of life (up to P15) and were expressed in reduced PC counts (dashed red right box). Concomitantly, the transcript levels of granular cell-type- and PC cell-specific markers were reduced (see result section). In addition, due to the reduced proliferative capacity after hyperoxia, it seems very likely that essential processes of cerebellar neurogenesis such as migration, proliferation, differentiation, maturation, dendritogenesis, and synaptogenesis are damaged by the early oxygen insult (red dashed lines, inhibition). Caffeine (dashed green left and right boxes) was able to reduce the effects of oxidative stress with respect to PCs, GPCs, and mitotic stages of GCs (green and blue lines, activation) but otherwise showed poor protective effects on granular cell neurogenesis (see Results). (b) Cellular players in cerebellar granular cell neurogenesis.

In the present study, we demonstrated the hyperoxic impairment of complex cerebellar neurogenesis of granular cells and essential calbindin expressing Purkinje cells with the neuronal and proliferative markers investigated here, as well as highlighting the protective effects of caffeine (see schematic illustration Figure 10). The number of PCs was greatly reduced in the oxygen-stressed postnatal cerebellum, and the GC precursors of the EGL were also drastically reduced at acute exposure times of 80% oxygen, with the peak of GC precursor proliferation at approximately P6 [29, 56]. This is then accompanied by severely reduced Shh transcription due to oxidative stress. Hyperoxia beginning at the time of birth also impaired cerebral proliferative capacity. This was reflected by reductions in factors important for cell cycle control, displayed by PCNA and *CycD2*. Postnatal between birth and P5, GPCs divide, increasing in number and thus enlarging the forming cerebellar lobules. Whereas after anatomical cerebellar development is complete, GPC divisions were downregulated until P15 as they exit the cell cycle, differentiate, and migrate [57]. The importance of Shh for cerebellar proliferation was also demonstrated by the work of Haldipur and colleagues [58]. Inhibition of Shh signaling resulted in a thinner EGL and also significantly minimized PCNA-positive cells and hindered further cell divisions [58]. Short-term exposure to high oxygen of newborn rats at P6 for 24 hours was also sufficient to sustainably reduce Shh and CycD2 through the influence of oxygen toxicity [59]. Our data revealed that GCPs are dramatically decreased in the oxidative stress response situation. The impaired proliferative capacity may affect subsequent processes, such as migration or further differentiation.

In this study, we examined several transcripts that are essential for further GPC development, survival, and GC migration from the EGL to the GCL. Hyperoxia for three days reduced the neurotrophic BDNF transcripts, but normal levels were established after recovery. Meanwhile, five-day hyperoxia first induced BNDF but then led to long-term downregulation. BDNF directly stimulates GPC migration [60] and BDNF forms a concentration gradient from EGL to GCL [61]. It would be possible that BDNF counterregulation at P5 under hyperoxia represents transcripts for proBDNF that are opposite in function to those of mature BDNF [62]. Thus, the absence of BDNF, only one of regulating factor of GC attraction [63], may affect the integration of GC into MCL. Coworkers of BDNF in GC-migration regulation are factors such as stromal cell-derived factor 1 (SDF1) [64, 65] or Ephrins [66]. NeuroD2 is essential for the survival of mature GC, while NeuroD1 assumes a key role in terminating the proliferation of GCPs to thereby initiate postmitotic GC migration [67, 68]. Depletion of NeuroD2 during the vulnerable postnatal period impaired GC survival and PC formation of synapses [68]. Alongside the decreased BDNF expression under postnatal hyperoxic conditions, we also demonstrated a dramatic reduction in NeuroD1 and NeuroD2 transcripts. Salero and Hatten examined the role of BDNF in *embryonic stem* cells, in which BDNF treatment of mitotic GCP induced postmitotic NeuroD expression [69]. Chd7 is highly expressed in GCs during cerebellar development and persists in the mature cerebellum, whereas PCs do not express Chd7 [70]. Mouse studies revealed the mechanism of cerebellar abnormalities caused by loss of function of Chd7. Similarly, heterozygous Chd7 mutation resulted in reduced expression of the key signaling molecule FGF8, which is essential for cerebellar development [70, 71], and FGF8 is able to induce postnatal GCP proliferation in the absence of Shh ligand [72]. In this study, both *Chd7* and *FGF8* were dramatically downregulated by oxygen toxicity, with no detectable changes after recovery to room air.

The initiation of cerebellar neurogenesis from tangential to radial migration along the Bergman glia of postmitotic GCs is mediated by Sema6a [73]. Functional disruption results in incomplete accumulation of mature GCs in the GCL [74]. The migration-regulating factor Lmx1*α* [31] is required for cerebellar formation [75] and postnatal development [76]. Cell type-specific markers were used to identify different cerebellar populations: Pax2 (GABAergic molecular layer interneurons [77, 78]), NeuN and Sox2 (differentiating GCPs [79, 80]), Prox1 (GCs [81]), and finally Syp (synaptogenesis, synaptic density [82, 83]). Essential to the underlying functions of the cerebellum are the highly organized circuits through the synaptic systems [84, 85]. All of these transcripts were detected to have decreased expression immediately after oxygen exposure and/or after recovery from exposure to high oxygen concentrations. In the context of cerebellar neurogenesis under the influence of oxygen toxicity, little is currently known about neuronal markers. When Sox2 is expressed in neuronal progenitors, it is postulated that it is downregulated during the start of differentiation and thus during the transition to postmitotic stages [86, 87]. In humans, cerebellar Sox2 expression is strong during pregnancy from week 20 to week 24 and decreases continuously until birth [88]. A rat injured model with cerebral ischemia undergoes the cerebellum specific and dynamic changes at the cellular level including cell proliferation and synaptogenesis. Jung et al. demonstrated a correlation between cerebellar proliferation capacity and expression of Syp [89].

A major factor that plays a predominant role in the molecular architecture of the developing cerebellum, not only for GC, is Pax6 [77]. Pax6 is expressed in granule progenitor cells (GCPs) that can differentiate into cerebellar nuclear neurons and postmitotic and mature granule cells as well as unipolar brush cells [90]. Pax6 regulates the migration and differentiation of GCs [90, 91]. Abnormalities of Pax6 are associated with aberrant organization of EGL or impaired cell cycle regulation [77, 90]. Nonetheless, these are all elementary players in cerebellar neurogenesis during a sensitive period of development. Impairments due to exogenous factors or physiological environmental changes suggest that changes may occur. We have already demonstrated that oxygen toxicity damages these types of processes in hippocampal neurogenesis in the 6-day-old rat model. Oxidative stress thereby reduced the expression of necessary transcription factors such as Pax6 and Prox1 and resulted in delayed neuronal maturation in the dentate gyrus [48, 92].

But ultimately, the results generated for relevant cerebral processes, such as migration or differentiation, or their mediators during cerebellar development were not surprising for hyperoxic injury but were completely unexpected for the hypothesized and expected caffeine effect.

Caffeine was able to reverse the hyperoxia-induced reduction of PCs and GPCs as well as the inhibition of proliferation at the cellular level, especially when oxygen had not acted on the developing cerebellum for more than three days. Caffeine already showed a short-term and transient better outcome for neurodevelopmental disorders in clinical studies of premature infants [42, 43, 93]. Considering the possible underlying mechanisms and properties of caffeine, antioxidant mechanisms of action and downstream probably anti-inflammatory as well as antiapoptotic effects are in the foreground [47–50, 92, 94]. Whether the mediation occurs directly via adenosine receptors [95] or indirectly through oxidative stress and/or ER stress reduction has not yet been conclusively clarified [48, 96, 97]. However, it has been clearly shown that caffeine has demonstrated this protection during vulnerable phases of brain development and it seems that caffeine has neuromodulatory properties [49, 98].

The complex interplay of diverse mechanisms during brain development and neuronal redox homeostasis determines neuronal susceptibility to oxidative stress in a dynamic dependence [99]. Oxidative stress is an inevitable factor in premature birth due to the transition from intrauterine hypoxia to extrauterine hyperoxia. Redox signaling is a critical aspect and is increasingly discussed in relation to therapeutic approaches in prematurity. Apart from caffeine-driven direct or indirect mechanisms, primarily the Nrf2/Nf*κ*B pathway seems to be important for intracellular transduction. Oxidative stress-associated genes, oxidative stress per se but also neuronal signal transduction, transcriptional regulation, and thus neuronal functions may be affected by methylxanthines [100].

Summarizing the generated caffeine effects on the hyperoxia-damaged postnatal cerebellum in this study, we see exclusively counteracting oxygen-damaging effects for the PCs at the cellular level and for the proliferating GCP. All downstream transcripts were hardly affected at all by caffeine. Sometimes, we detected a positive effect after recovery to room air, mainly when the oxygen insult was only three days long. Vogel et al. postulated that there is a constant ratio of GCs for each PC, suggesting that the number of GCs is regulated by the number and duration of Shh secretion by PCs [101]. Under hyperoxia, both PC number and, in correlation with this, the amount of Shh are reduced, from which the reduction of Pax6-positive and proliferating GCP could be explained.

Pax6 itself has neurogenic functions and is closely associated with the modulatory capabilities of the cell cycle; initiating the exit from the cell cycle to begin differentiation and modulate downstream transcripts directly or indirectly [102–104]. Downregulated Pax6 could be rescued by caffeine after three days of hyperoxia, which was maintained until P15. Five-day hyperoxia also reduced Pax6, and caffeine brought it to normoxia levels. Downstream transcripts did not benefit and remained decreased under hyperoxia with caffeine, but this consistently affected factors also expressed by GCP. Mature neuronal markers were more likely to be affected after prolonged injury, possibly based on the Pax6 peak at P6 [104].

Aspects that need to be mentioned in the discussion are the influence of caffeine on other receptors, such as interactions with *γ*-aminobutyric acid type A receptors; the influence of kinetic/dynamic expression profiles of adenosine receptors and the adenosinergic system during postnatal brain development [105]; varying oxygen environments in neonatal brain areas, which may alter the caffeine pharmacodynamics and even reverse the benefits of caffeine [106, 107]; and dedifferentiation of GCPs into astroglia cells [108], as well as a systemic inflammation from oxidative stress or even caffeine, which is known to have adverse effects on the developing brain, and which, coupled with a proinflammatory cytokine profile in preterm infants treated with higher doses of caffeine, may be responsible for the deleterious effects on the cerebellum [109, 110].

Similarly, caffeine exhibited inappropriate side effects in clinical and preclinical studies, as well as impaired dendrite length of PCs as well as transcripts mainly of postmitotic and differentiated GCs detected in this study. Early high-dose caffeine demonstrated a higher incidence of cerebellar injury and early motor performance [109]. High caffeine citrate increased total dendritic length and arborization of layer III pyramidal neurons of the prefrontal cortex at P35 and P70 of newborn rat pups when administrated at P1 to P12 [111]. Decreased cerebellar weight and increased saturated fatty acid concentration of the cerebellum were detected after caffeine citrate administration to newborn rats for ten postnatal days [112]. Moderate caffeine administrated to rat pups from P2 until P6 decreased adenosine receptor subtype A1 binding in the molecular layer of cerebellum [113].

PCs and GCs are closely related during cerebellar neurogenesis, and Pax6 plays a central role in regulating differentiation processes. A reduced GCP proliferation capacity would lead one to expect a reduced number of mature GCs, as shown by Iskusnykh and colleagues in preterm pigs compared to term pigs [114].

The survival of premature infants with good quality of life is in focus, and an important aspect is the controlled therapy with oxygen. Due to the imbalance of oxidative stress and the underdeveloped antioxidant enzyme system, the effects of oxygen toxicity as well as the use of drugs on cerebellar development are almost inevitable [17, 59, 115, 116]. Impaired GC neurogenesis is able to reshape and modulate downstream targets, which can lead to cerebellar downstream circulatory dysfunction [117]. Cognitive-motor, social-emotional, and linguistic deficits can be attributed to disorders of the cerebellum during this dynamic phase [26, 118, 119].

Neuron-glia communication is required for brain functioning and essential during early neurodevelopment. PC loss and GCP reduction were recovered with caffeine, but the trailed worse effect of caffeine on hyperoxia-damaged GC-relevant transcripts needs further investigation, as caffeine itself did not affect GCPs and PCs.

Since caffeine has so far demonstrated good therapeutic effects in the clinical setting of preterm infants as well as in in vitro and in vivo studies, the protective properties predominate, although further studies on caffeine with regard to possible pleiotropic effects appear necessary.

Preterm birth disrupts cerebellar development. Systemic oxidative stress caused by premature birth appears to be a major contributor to damage to neuronal developmental stages. Here, we were able to show that oxidative stress could significantly damage the cerebellar neurogenesis in the postnatal rat brain with regard to GCPs and PCs. The supposed cellular neuronal damage is reversible, whereby it subjectively cancels itself again and can be rationally more effective through the treatment with caffeine. Downstream transcripts classified for migration and differentiation of postmitotic GCs appear to be irreversibly reduced. The hypothesis that caffeine had unlimited neuroprotective effects under oxidative stress in the cerebellum could not be confirmed in comparison to other brain regions. The protection of neurons that we observed in the postnatal hyperoxic-injured hippocampus after early-life caffeine exposure was not seen in the cerebellum [48, 49]. This difference might be attributable to different oxygen environments, varying levels of vulnerability dependent on the developmental stage, varying transcriptional regulation of redox-sensitive response to oxygen toxicity, varying of receptor density, and/or proinflammatory insult compared to other brain regions.

With regard to the discussion about the preventive use of caffeine in ventilated premature infants, the restrictions that caffeine initiates under control conditions must be taken into account. Differentiated clinical observations on the long-term outcome seem essential to ensure the safe use of this standard therapeutic agent.

## 5. Conclusions

We show that oxidative stress can damage the cerebellar neurogenesis in the postnatal rat brain with regard to GCPs and PCs. The cellular neuronal damage is partly reversible and rescued by caffeine. However, downstream transcripts important for migration and differentiation of postmitotic GCs are irreversibly reduced by oxidative stress, and this effect is not rescued by caffeine. The protection of neurons that we observed in the postnatal hyperoxic-injured hippocampus after early-life caffeine exposure was not seen in the cerebellum. This difference might be attributable to different oxygen environments, varying levels of vulnerability dependent of developmental stage, varying transcriptional regulation of redox-sensitive response to oxygen toxicity, and varying of receptor density and/or proinflammatory insult compared to other brain regions.

## Figures and Tables

**Table 1:** Sequences of oligonucleotides.

	Oligonucleotide sequence 5′-3′	Accession No.
*BDNF*		
Forward	TCAGCAGTCAAGTGCCTTTGG	NM_012513.4
Reverse	CGCCGAACCCTCATAGACATG	
Probe	CCTCCTCTGCTCTTTCTGCTGGAGGAATACAA	
*Calb1*		
Forward	CGACGCTGATGGAAGTGGTT	NM_031984.2
Reverse	TCCAATCCAGCCTTCTTTCG	
Probe	AAGGAAAGGAGCTGCAGAA	
*Chd7*		
Forward	CAAGCTTCTGGAGGGACTGAA	NM_001107906.2
Reverse	AAGAGCTCCTCCACAGTGTTCTG	
Probe	TGGAACACAAAGTGCTGC	
*CycD2*		
Forward	CGTACATGCGCAGGATGGT	NM_199501.1
Reverse	AATTCATGGCCAGAGGAAAGAC	
Probe	TGGATGCTAGAGGTCTGTGA	
*FGF8*		
Forward	CCTTCGCAAAGCTCATTGTG	NM_133286.1
Reverse	CCCTTCTTGTTCATGCAGATGTAC	
Probe	TTTTGGAAGCAGAGTCCGAGT	
*HPRT*		
Forward	GGAAAGAACGTCTTGATTGTTGAA	NM_012583.2
Reverse	CCAACACTTCGAGAGGTCCTTTT	
Probe	CTTTCCTTGGTCAAGCAGTACAGCCCC	
*Lmx1α*		
Forward	ACCACTCAGCAGAGGAGAGCAT	NM_001105967.2
Reverse	TGTCTCCGCAGCCAGAGTCT	
Probe	AAGTATCCTCCAAGCCCT	
*NeuroD1*		
Forward	TCAGCATCAATGGCAACTTC	NM_019218.2
Reverse	AAGATTGATCCGTGGCTTTG	
Probe	TTACCATGCACTACCCTGCA	
*NeuroD2*		
Forward	TCTGGTGTCCTACGTGCAGA	NM_019326.1
Reverse	CCTGCTCCGTGAGGAAGTTA	
Probe	TGCCTGCAGCTGAACTCTC	
*NeuN*		
Forward	GCTGAATGGGACGATCGTAGAG	NM_001134498.2
Reverse	CATATGGGTTCCCAGGCTTCT	
Probe	AGGTCAATAATGCCACGGC	
*Pax2*		
Forward	GTACTACGAGACTGGCAGCATCAA	NM_001106361.1
Reverse	TCGGGTTCTGTCGCTTGTATT	
Probe	CCAAAGTGGTGGACAAGA	
*Pax6*		
Forward	TCCCTATCAGCAGCAGTTTCAGT	NM_013001.2
Reverse	GTCTGTGCGGCCCAACAT	
Probe	CTCCTCCTTTACATCGGGTT	
*Prox1*		
Forward	TGCCTTTTCCAGGAGCAACTAT	NM_001107201.1
Reverse	CCGCTGGCTTGGAAACTG	
Probe	ACATGAACAAAAACGGTGGC	
*Sema6a*		
Forward	GCCATTGATGCGGTCATTTA	NM_001108430.2
Reverse	GTACGGCTCTTTCAACCACTTTG	
Probe	CTTGGAGACAGTCCTACC	
*Shh*		
Forward	GCTGATGACTCAGAGGTGCAAA	NM_017221.1
Reverse	CCTCAGTCACTCGAAGCTTCACT	
Probe	CAAGTTAAATGCCTTGGCCA	
*Sox2*		
Forward	ACAGATGCAGCCGATGCA	NM_001109181.1
Reverse	GGTGCCCTGCTGCGAGTA	
Probe	CAGTACAACTCCATGACCAG	
*Syp*		
Forward	TTCAGGCTGCACCAAGTGTA	NM_003179.3
Reverse	TTCAGCCGACGAGGAGTAGT	
Probe	AGGGGGCACTACCAAGATCT	

## Data Availability

The data used to support the findings of this study are available from the corresponding author upon request. The analyzed data used to create the graphs and statistical evaluation are attached in the supplemented material of this work.
