# Supplementary material for: The Conflicting Role of Caffeine Supplementation on Hyperoxia-Induced Injury on the Cerebellar Granular Cell Neurogenesis of Newborn Rats
Source: Oxid Med Cell Longev. 2022 May 31;2022:5769784. doi: 10.1155/2022/5769784 (PMC9175096; doi:10.1155/2022/5769784)
Supplement: Supplementary Materials — All basic data of the created diagrams are available in the following supplementary tables (Tables S1–S6). [file 5769784.f6.zip › 5769784.f1.docx]

**Table S-1** Quantitation of Calbindin positive cells and Purkinje cell dendrite length after oxygen-induced cerebellar neurotoxicity with/without caffeine

| **hyperoxia**  **caffeine** | **-**  **-** | **+**  **-** | **-**  **+** | **+**  **+** | **hyperoxia**  **caffeine** | **-**  **-** | **+**  **-** | **-**  **+** | **+**  **+** |
| --- | --- | --- | --- | --- | --- | --- | --- | --- | --- |
| **P3** | | | | | **P3_P15** | | | | |
| **Calbindin+**  **Dendrite length** | 100±7.0  100±2.2 | **^b^**66±4.7  **^b^**79±4.6 | 100±5.8  101±4.4 | **^f^**100±9.0  **^f^**100±5.5 | **Calbindin+**  **Dendrite length** | 100±8.8  100±9.9 | 108±6.7  87±5.6 | 95±5.4  87±4.1 | 104±9.9  85±4.8 |
| **P5** | | | | | **P5_P15** | | | | |
| **Calbindin+**  **Dendrite length** | 100±6.0  100±4.6 | **^b^**75±2.8  **^b^**79±2.6 | 102±3.4  **^b^**80±2.9 | **^e^**93±4.1  90±3.4 | **Calbindin+**  **Dendrite length** | 100±1.7  100±2.5 | **^c^**70±5.2  95±5.0 | 86±4.3  100±7.6 | **^e^**92±3.9  110±7.1 |

Data are normalized to the level of rat pups exposed to normoxia at each time point (control 100 %, white bars) and the 100 % values are 1.4 (P3), 2.8 (P3_P15), 1.4 (P5), and 2.5 (P5_P15) length of molecular layer, or 14 (P3), 7.4 (P3_P15), 8.9 (P5), and 7.2 (P5_P15) cells per regions of lobules, respectively. Data expressed as % of control as mean ± SEM with n = 6-8/ group. ^a^ p < 0.05, ^b^ p < 0.01, ^c^ p < 0.001, ^d^ p < 0.0001 vs. control; ^e^p < 0.05, ^f^p < 0.01, ^g^p < 0.001 vs. hyperoxia (ANOVA, Bonferroni's *post hoc* test; Kruskal-Wallis, Dunn´s *post hoc* test).
