# Supplementary material for: The Conflicting Role of Caffeine Supplementation on Hyperoxia-Induced Injury on the Cerebellar Granular Cell Neurogenesis of Newborn Rats
Source: Oxid Med Cell Longev. 2022 May 31;2022:5769784. doi: 10.1155/2022/5769784 (PMC9175096; doi:10.1155/2022/5769784)
Supplement: Supplementary Materials — All basic data of the created diagrams are available in the following supplementary tables (Tables S1–S6). [file 5769784.f6.zip › 5769784.f2.docx]

**Table S-2** Quantitation of Pax6 and PCNA positive cells after oxygen-induced cerebellar neurotoxicity with/without caffeine

| **hyperoxia**  **caffeine** | **-**  **-** | **+**  **-** | **-**  **+** | **+**  **+** | **hyperoxia**  **caffeine** | **-**  **-** | **+**  **-** | **-**  **+** | **+**  **+** |
| --- | --- | --- | --- | --- | --- | --- | --- | --- | --- |
| **P3** | | | | | **P3_P15** | | | | |
| **Pax6+**  **PCNA** | 100±5.0  100±7.2 | **^b^**73±3.3  **^c^**62±2.5 | 82±5.1  89±7.8 | **^d^**106±12.0  **^e^**99±4.8 | **Pax6+**  **PCNA** | 100±5.9  100±2.8 | 120±15.0  **^b^**75±0.6 | 104±4.3  95±8.5 | **^a^**130±7.1  **^d^**106±6.5 |
| **P5** | | | | | **P5_P15** | | | | |
| **Pax6+**  **PCNA** | 100±5.2  100±8.0 | **^b^**72±2.9  **^b^**68±4.7 | 88±7.1  82±4.0 | 81±1.7  **^b^**70±2.9 | **Pax6+**  **PCNA** | 100±7.6  100±6.4 | 109±10.0  129±13.1 | 111±9.0  103±8.0 | 122±7.3  126±5.0 |

Data are normalized to the level of rat pups exposed to normoxia at each time point (control 100 %, white bars) and the 100 % values are 38 (P3), 54.4 (P3_P15), 46.6 (P5), and 41.3 (P5_P15) Pax6+ cells per regions of lobules, or 33 (P3), 37.1 (P3_P15), 65.7 (P5), and 22.3 (P5_P15) PCNA+ cells per regions of lobules, respectively. Data expressed as % of control as mean ± SEM with n = 6-8/ group. ^a^ p < 0.05, ^b^ p < 0.01, ^c^ p < 0.001 vs. control; ^d^p < 0.05, ^e^p < 0.01 vs. hyperoxia (ANOVA, Bonferroni's *post hoc* test; Kruskal-Wallis, Dunn´s *post hoc* test; Brown-Forsythe, Dunnett´s *post hoc* test).
