# Supplementary material for: The Conflicting Role of Caffeine Supplementation on Hyperoxia-Induced Injury on the Cerebellar Granular Cell Neurogenesis of Newborn Rats
Source: Oxid Med Cell Longev. 2022 May 31;2022:5769784. doi: 10.1155/2022/5769784 (PMC9175096; doi:10.1155/2022/5769784)
Supplement: Supplementary Materials — All basic data of the created diagrams are available in the following supplementary tables (Tables S1–S6). [file 5769784.f6.zip › 5769784.f3.docx]

**Table S-3** Quantitation of granule cell precursor -associated mRNA expression after oxygen-induced cerebellar neurotoxicity with/without caffeine

| **hyperoxia**  **caffeine** | **-**  **-** | **+**  **-** | **-**  **+** | **+**  **+** | **hyperoxia**  **caffeine** | **-**  **-** | **+**  **-** | **-**  **+** | **+**  **+** |
| --- | --- | --- | --- | --- | --- | --- | --- | --- | --- |
| **P3** | | | | | **P3_P15** | | | | |
| *FGF8*  *CycD2*  *Sema6a*  *Lmx1α* | 100±10.0  100±7.3  100±8.1  100±5.8 | 107±7.7  **^c^**59±4.8  83±7.0  **^d^**54±4.5 | 107±7.5  80±9.3  124±8.3  76±8.2 | 75±12.9  **^b^**60±3.3  80±5.9  **^b^**71±6.2 | *FGF8*  *CycD2*  *Sema6a*  *Lmx1α* | n.d.  100±3.4  100±5.1  100±5.7 | n.d.  **^b^**71±5.8  84±5.7  88±9.2 | n.d.  87±5.4  87±5.8  **^b^**61±9.6 | n.d.  **^a,f^**125±5.7  90±10.3  **^a^**65±6.4 |
| **P5** | | | | | **P5_P15** | | | | |
| *FGF8*  *CycD2*  *Sema6a*  *Lmx1α* | 100±6.8  100±5.2  100±8.4  100±6.2 | **^a^**71±4.2  **^d^**47±7.4  **^b^**58±5.4  125±4.9 | 196±30.4  **^a^**132±7.7  85±7.9  98±6.6 | **^b^**53±7.3  **^d^**50±7.8  **^a^**58±5.6  **^e^**90±9.0 | *FGF8*  *CycD2*  *Sema6a*  *Lmx1α* | n.d.  100±4.1  100±7.6  100±7.0 | n.d.  **^b^**67±8.5  112±13.2  118±2.0 | n.d.  92±6.5  104±7.1  **^b^**144±11.1 | n.d.  90±3.9  113±10.0  **^c^**144±5.8 |

Data are normalized to the level of rat pups exposed to normoxia at each time point (control 100 %, white bars). Data expressed as % of control as mean ± SEM with n = 6-8/ group. ^a^ p < 0.05, ^b^ p < 0.01, ^c^ p < 0.001, ^d^ p < 0.0001 vs. control; ^e^p < 0.0001 vs. hyperoxia (ANOVA, Bonferroni's *post hoc* test; Kruskal-Wallis, Dunn´s *post hoc* test; Brown-Forsythe, Dunnett´s *post hoc* test).
