# Supplementary material for: The Conflicting Role of Caffeine Supplementation on Hyperoxia-Induced Injury on the Cerebellar Granular Cell Neurogenesis of Newborn Rats
Source: Oxid Med Cell Longev. 2022 May 31;2022:5769784. doi: 10.1155/2022/5769784 (PMC9175096; doi:10.1155/2022/5769784)
Supplement: Supplementary Materials — All basic data of the created diagrams are available in the following supplementary tables (Tables S1–S6). [file 5769784.f6.zip › 5769784.f4.docx]

**Table S-4** Quantitation of granule cell precursor- and/or Purkinje cell-associated mRNA expression after oxygen-induced cerebellar neurotoxicity with/without caffeine

| **hyperoxia**  **caffeine** | **-**  **-** | **+**  **-** | **-**  **+** | **+**  **+** | **hyperoxia**  **caffeine** | **-**  **-** | **+**  **-** | **-**  **+** | **+**  **+** |
| --- | --- | --- | --- | --- | --- | --- | --- | --- | --- |
| **P3** | | | | | **P3_P15** | | | | |
| *BDNF*  *Calb1*  *Shh* | 100±4.0  100±2.7  100±5.6 | **^b^**80±1.7  **^d^**68±2.0  **^c^**67±3.1 | **^a^**79±5.7  **^d^**65±3.1  **^b^**72±6.0 | **^b^**69±5.5  **^d^**76±2.9  **^c^**63±5.5 | *BDNF*  *Calb1*  *Shh* | 100±5.5  100±10.0  100±6.9 | 98±6.7  87±7.6  109±7.4 | 95±5.1  88±6.0  102±8.0 | 98±7.5  **^e^**148±11.4  111±8.7 |
| **P5** | | | | | **P5_P15** | | | | |
| *BDNF*  *Calb1*  *Shh* | 100±4.9  100±8.4  100±6.0 | **^d^**133±3.5  **^b^**58±5.4  100±6.9 | **^c^**70±6.0  103±11.4  **^a^**77±6.7 | **^f^**105±3.4  **^a^**59±8.0  77±4.2 | *BDNF*  *Calb1*  *Shh* | 100±4.6  100±6.3  100±3.3 | **^c^**60±4.3  **^b^**60±9.0  **^b^**69±5.0 | **^a^**73±4.5  91±6.2  88±6.6 | **^e^**95±3.7  82±5.3  **^e^**97±5.5 |

Data are normalized to the level of rat pups exposed to normoxia at each time point (control 100 %, white bars). Data expressed as % of control as mean ± SEM with n = 6-8/ group. ^a^ p < 0.05, ^b^ p < 0.01, ^c^ p < 0.001, ^d^ p < 0.0001 vs. control; ^e^p < 0.01, ^f^p < 0.001 vs. hyperoxia (ANOVA, Bonferroni's *post hoc* test; Kruskal-Wallis, Dunn´s *post hoc* test; Brown-Forsythe, Dunnett´s *post hoc* test).
