# Supplementary material for: The Conflicting Role of Caffeine Supplementation on Hyperoxia-Induced Injury on the Cerebellar Granular Cell Neurogenesis of Newborn Rats
Source: Oxid Med Cell Longev. 2022 May 31;2022:5769784. doi: 10.1155/2022/5769784 (PMC9175096; doi:10.1155/2022/5769784)
Supplement: Supplementary Materials — All basic data of the created diagrams are available in the following supplementary tables (Tables S1–S6). [file 5769784.f6.zip › 5769784.f5.docx]

**Table S-5** Quantitation of granule cell precursor- and granule cell-associated mRNA expression after oxygen-induced cerebellar neurotoxicity with/without caffeine

| **hyperoxia**  **caffeine** | **-**  **-** | **+**  **-** | **-**  **+** | **+**  **+** | **hyperoxia**  **caffeine** | **-**  **-** | **+**  **-** | **-**  **+** | **+**  **+** |
| --- | --- | --- | --- | --- | --- | --- | --- | --- | --- |
| **P3** | | | | | **P3_P15** | | | | |
| *Pax6*  *Chd7*  *NeuroD1*  *NeuN*  *Prox1* | 100±8.3  100±5.6  100±6.8  100±5.5  100±8.5 | **^c^**54±4.7  **^d^**56±4.5  **^d^**46±7.0  **^d^**55±2.7  87±6.6 | **^b^**63±8.6  **^a^**74±8.5  **^c^**55±8.1  84±7.3  **^a^**72±7.7 | **^c^**54±3.8  **^d^**54±4.6  **^c^**51±4.5  **^c^**65±4.1  **^b^**64±5.2 | *Pax6*  *Chd7*  *NeuroD1*  *NeuN*  *Prox1* | 100±4.7  100±9.0  100±5.7  100±9.7  100±5.6 | **^b^**63±8.6  76±9.0  **^c^**61±8.9  **^c^**45±6.8  91±7.0 | **^b^**60±7.0  96±6.3  **^b^**65±3.0  **^b^**54±8.0  85±6.4 | **^e^**94±7.8  86±13.3  78±4.9  **^f^**90±6.2  **^a,g^**131±7.8 |
| **P5** | | | | | **P5_P15** | | | | |
| *Pax6*  *Chd7*  *NeuroD1*  *NeuN*  *Prox1* | 100±2.9  100±5.0  100±6.0  100±7.7  100±3.8 | **^b^**50±8.6  **^b^**62±5.3  **^c^**40±8.2  **^b^**61±2.8  95±6.7 | **^b^**159±9.4  **^b^**145±11.2  107±8.9  **^a^**64±4.4  **^c^**73±1.6 | **^a^**61±9.0  **^a^**69±8.1  **^c^**47±10.0  **^c^**60±3.2  **^b,e^**61±7.1 | *Pax6*  *Chd7*  *NeuroD1*  *NeuN*  *Prox1* | 100±5.9  100±9.1  100±4.8  100±6.5  100±4.2 | 97±12.6  129±9.3  **^b^**132±4.8  **^c^**161±11.0  94±3.1 | 125±5.6  101±1.8  **^d^**179±7.4  **^a^**136±8.1  99±6.6 | 94±6.7  96±9.0  **^f^**102±3.5  **^g^**106±8.8  110±5.1 |

Data are normalized to the level of rat pups exposed to normoxia at each time point (control 100 %, white bars). Data expressed as % of control as mean ± SEM with n = 6-8/ group. ^a^ p < 0.05, ^b^ p < 0.01, ^c^ p < 0.001, ^d^ p < 0.0001 vs. control; ^e^p < 0.05, ^f^p < 0.01, ^g^p < 0.001 vs. hyperoxia (ANOVA, Bonferroni's *post hoc* test; Kruskal-Wallis, Dunn´s *post hoc* test; Brown-Forsythe, Dunnett´s *post hoc* test).
