# Supplementary material for: The Conflicting Role of Caffeine Supplementation on Hyperoxia-Induced Injury on the Cerebellar Granular Cell Neurogenesis of Newborn Rats
Source: Oxid Med Cell Longev. 2022 May 31;2022:5769784. doi: 10.1155/2022/5769784 (PMC9175096; doi:10.1155/2022/5769784)
Supplement: Supplementary Materials — All basic data of the created diagrams are available in the following supplementary tables (Tables S1–S6). [file 5769784.f6.zip › 5769784.f6.docx]

**Table S-6** Quantitation of postmitotic and mature granule cell-associated mRNA expression after oxygen-induced cerebellar neurotoxicity with/without caffeine

| **hyperoxia**  **caffeine** | **-**  **-** | **+**  **-** | **-**  **+** | **+**  **+** | **hyperoxia**  **caffeine** | **-**  **-** | **+**  **-** | **-**  **+** | **+**  **+** |
| --- | --- | --- | --- | --- | --- | --- | --- | --- | --- |
| **P3** | | | | | **P3_P15** | | | | |
| *Pax2*  *NeuroD2*  *Syp*  *Sox2* | 100±5,2  100±5,5  100±6,7  100±6,7 | **^b^**48±5,7  111±8,5  **^a^**73±2,7  103±1,6 | 78±10,4  120±12,1  **^b^**65±1,6  112±8,5 | 81±12,8  125±8,5  **^b^**65±3,0  90±4,0 | *Pax2*  *NeuroD2*  *Syp*  *Sox2* | 100±5,7  100±6,1  100±7,1  100±7,3 | **^b^**67±5,0  114±13,1  96±7,3  123±5,8 | 101±4,6  99±13,0  81±6,2  120±5,9 | 77±13,1  79±6,3  105±8,7  **^c^**140±5,3 |
| **P5** | | | | | **P5_P15** | | | | |
| *Pax2*  *NeuroD2*  *Syp*  *Sox2* | 100±4,8  100±5,6  100±4,2  100±5,2 | **^b^**64±4,4  **^d^**37±5,2  91±3,4  96±1,6 | 91±9,5  **^c^**59±8,7  **^b^**81±3,3  93±6,7 | **^c^**52±8,7  **^d^**31±4,0  90±4,2  **^a,f^**78±2,8 | *Pax2*  *NeuroD2*  *Syp*  *Sox2* | 100±4,1  100±4,2  100±5,2  100±3,1 | 95±9,1  **^d^**147±5,1  96±7,7  **^b^**73±4,7 | **^a^**131±6,6  **^b^**183±12,9  110±6,1  100±6,0 | 114±9,2  **^e^**112±4,9  103±6,4  **^f^**108±4,0 |

Data are normalized to the level of rat pups exposed to normoxia at each time point (control 100 %, white bars). Data expressed as % of control as mean ± SEM with n = 6-8/ group. ^a^ p < 0.05, ^b^ p < 0.01, ^c^ p < 0.001, ^d^ p < 0.0001 vs. control; ^e^p < 0.01, ^f^p < 0.001 vs. hyperoxia (ANOVA, Bonferroni's *post hoc* test; Kruskal-Wallis, Dunn´s *post hoc* test; Brown-Forsythe, Dunnett´s *post hoc* test).
